# HBV and HCV Co-Infection in Chinese Newly Diagnosed HIV+ Subjects in 2015 and 2023: A Cross-Sectional Study

**DOI:** 10.3390/pathogens13050367

**Published:** 2024-04-29

**Authors:** Fangyuan Li, Yi Feng, Xiu Liu, Jingjing Hao, Dong Wang, Hongping Hu, Chang Song, Jing Hu, Quanbi Zhao, Hua Liang, Lingjie Liao, Yuhua Ruan, Hui Xing

**Affiliations:** National Key Laboratory of Intelligent Tracking and Forecasting for Infectious Diseases, National Center for AIDS/STD Control and Prevention, Chinese Center for Disease Control and Prevention, Beijing 102206, China; lifangyuan_0522@163.com (F.L.); fengyi@chinaaids.cn (Y.F.); liuxiu210508@163.com (X.L.); haojingjing@chinaaids.cn (J.H.); wangdong19971229@163.com (D.W.); harolinee@163.com (H.H.); songchang723@chinaaids.cn (C.S.); hujing@chinaaids.cn (J.H.); qbzhao@chinaaids.cn (Q.Z.); lianghua@chinaaids.cn (H.L.); liaolj@chinaaids.cn (L.L.); ruanyuhua92@chinaaids.cn (Y.R.)

**Keywords:** HIV, HBV, HCV, infection route, co-infection

## Abstract

With shared routes of transmission, HBV and HCV co-infection are estimated to occur more in subjects with HIV. This study aimed to characterize and describe the prevalence of HBV and HCV co-infections in a cohort of newly diagnosed HIV+ subjects living in China. We conducted a cross-sectional study among newly diagnosed HIV+ subjects aged 18–100 who participated in surveys on the national HIV molecular epidemiology in 2015 and 2023. (The epidemiological table survey is located in the national database alongside serologic testing). The chi-square test was used to identify changes in infections between the studying populations in 2015 and 2023, and conditional logistic regression models were fit to identify risk factors for each co-infection. Among the 11,024 newly diagnosed HIV+ subjects who were surveyed (n = 4501 in 2015; n = 6523 in 2023), the prevalence of HBV, HCV, and HBV/HCV in 2023 was lower than that in 2015, respectively. No decrease was observed in HCV co-infection in men who had sex with men (MSM) in North China, Northeast China, and East China. Increasing recognition among those at high risk of heterosexual transmission and those with low educational backgrounds is paramount to the prevention and control of HIV/HBV/HCV infections.

## 1. Introduction

In 2023, there were 1.26 million people living with HIV in China [1]. According to modeling studies, China has the highest rate of hepatitis B virus (HBV) and hepatitis C virus (HCV) infections [2,3]. The number of HBV-positive individuals in China reached 79,747,000 in 2022, while the number of HCV-positive individuals in China reached 2.3 million in 2020, and 9.487 million people have HCV worldwide [2,3]. In 2016, the WHO set the target of eliminating HIV, HBV, and HCV as a public health threat by 2030. In 2021, nine organizations in China, including the National Health and Health Commission of the People’s Republic of China, released the “Work Program for Action to Eliminate Public Health Hazards of Hepatitis C (2021–2030)”. Since then, the prevention of HIV, HBV, and HCV has attracted more and more attention. Additionally, HIV/HBV and HIV/HCV co-infections were reported to accelerate HIV disease progression and high antiretroviral therapy-related deaths compared to mono-infected individuals [4,5,6]. The early combining of diagnosis and treatment, risk behaviors and reduction strategies, and the hepatitis B vaccine are effective to eliminate HBV and HCV in people living with HIV [7,8,9].

In this study, to elucidate the current status of HIV co-infection with HBV and/or HCV, a comparison was made between populations of newly diagnosed HIV-infected individuals in 2015 and 2023 who were recruited for the surveys of national HIV molecular epidemiology. These data provide a reference for the prevention and treatment of HIV/HBV and HIV/HCV co-infections.

## 2. Materials and Methods

### 2.1. Study Location and Methodology for the Survey

A total of 11,024 people who were newly diagnosed HIV+ subjects from 31 provinces in China were collected by sampling in 2015 (n = 4501) and in 2023 (n = 6523). A cross-sectional survey method with a two-stage cluster design was used for sampling in each province. Based on the average reported cases of HIV-infected subjects reported over the last three years, each province was divided into the following three levels: high prevalence, medium prevalence, and low prevalence cities. At least two prefectural-level cities were chosen as the sampling area from cities of each prevalence level. Also, the minimum number of blood samples was determined based on the average reported cases of HIV-infected patients over the past three years. Each sampled prefecture-level city was included in the group according to the order of newly diagnosed HIV-infected individuals. Higher sampling ratios were used for the provinces and autonomous regions, with fewer cases were reported to ensure sample sufficiency. An epidemiologic questionnaire for HIV cases, including age, gender, ethnicity, literacy, marital status, reporting province, HIV infection route, and first-time CD4+ T lymphocyte count, was used to collect demographic characteristics.

### 2.2. Subjects of the Study

Individuals newly diagnosed with HIV in 2015 and 2023 were recruited. The inclusion criteria of the study subjects were as follows: (1) age ≥ 18 years; (2) HIV+ subjects who had not received any antiviral treatment; (3) all survey participants had to have a card ID; (4) the first CD4+ T lymphocyte count (CD4) had to be greater than 200 (cells/μL) to exclude AIDS; and (5) subjects had to complete informed consent forms. (The ethics approval number is X140617334).

### 2.3. Laboratory Tests

Plasma samples were collected by the local Center for Disease Control and Prevention (CDC) laboratory personnel and transported to the National Center for AIDS/STD Control and Prevention, Chinese Center for Disease Control and Prevention (China CDC) for testing. CD4+ T lymphocyte count testing is performed by laboratories that have passed the national quality control examination. Qualitative detections of HBsAg and anti-HCV were performed at the first diagnosis of HIV infection using a commercially available enzyme-linked immunosorbent kit (Wantai Biological Pharmacy, Beijing, China) following the manufacturer’s instructions.

### 2.4. Statistical Analysis

The data were arranged and analyzed using SAS 9.4 and GraphPad Prism 8.0.2 software. The χ2 test was used to compare the co-infection of HBV and HCV among HIV-infected individuals with different routes of infection and different areas in 2015 and 2023 to characterize the overall circumstances of the survey respondents. The factors influencing the combined HBV and HCV infections among newly diagnosed HIV+ subjects in 2023 were examined using a one-way logistic regression model. The independent variables that showed statistical significance in the one-way analysis were then analyzed using a multifactorial logistic regression model, and the independent variables were stepwise-regression-screened using a 0.05 cutoff. A two-sided test was run with a *p* < 0.05 test level.

## 3. Results

### 3.1. Demographic Characteristics and Subtype Profile of the Study Population

Among the 11,024 (4501 in 2015 and 6523 in 2023) newly diagnosed HIV patients in China, 39.8% (4386/11,024) were aged 18~34 years; 77.4% (8535/11,024) were male; 84.8% (9352/11,024) were Chinese Han; and 29.9% (3293/11,024) were elementary school or illiterate; 42.2% (4652/11,024) were married or cohabiting; 58.3% (6431/11,024) were infected as a result of heterosexual transmission; 43.6% (4808/11,024) had an initial CD4+ T lymphocyte count of 200–349; and 28.5% (3146/11,024) and 22.6% (2496/11,024) of the subjects were newly diagnosed in Southwest China and East China, respectively (Table 1).

### 3.2. Prevalence of HIV/HBV Co-Infection in China

**Figure 1:**
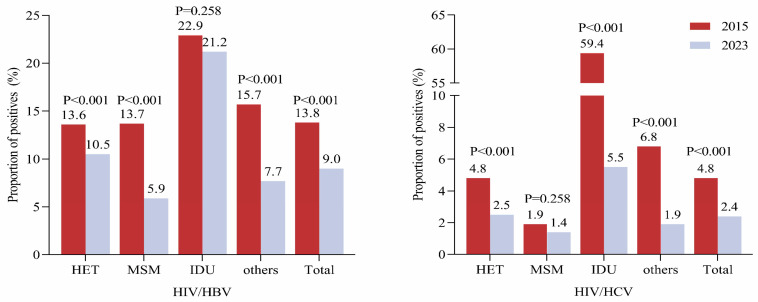
HBV (**left**) and HCV (**right**) co-infection in newly diagnosed HIV-infected patients with different transmission routes in 2015 (red) and 2023 (grey). Note: others include modes of transmission such as mother-to-child transmission and lack of information. *p* values were calculated using the chi-square test. *p* values < 0.05 are statistically significant.

**Figure 2:**
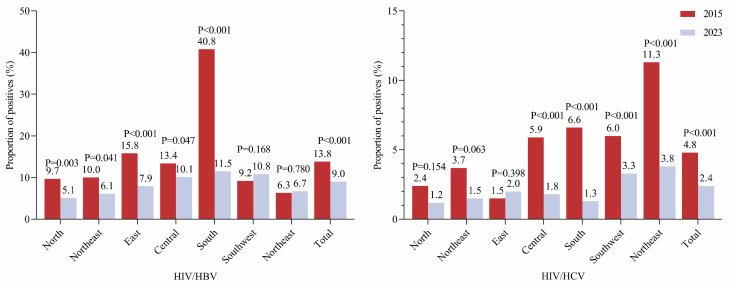
HBV and HCV co-infection in newly diagnosed HIV-infected patients in different regions in 2015 (red) and 2023 (grey). Note: The colors represent 2015 or 2023, and *p* values were calculated using the chi-square test. *p* values < 0.05 are statistically significant. North provinces: Beijing (BJ), Tianjin (TJ), Hebei (HE), Shanxi (SX), and Inner Mongolia Autonomous Region (NM). Northeast provinces: Liaoning (LN), Jilin (JL), and Heilongjiang (HLJ). Eastern provinces: Shanghai (SH), Jiangsu (JS), Zhejiang (ZJ), Jiangxi (JX), Anhui (AH), Fujian (FJ), and Shandong (SD). Central provinces: Henan (HA), Hubei (HB), and Hunan (HN). Southern provinces: Guangdong (GD), Guangxi Zhuang Autonomous Region (GX), and Hainan (HI). Southwest provinces: Chongqing (CQ), Sichuan (SC), Guizhou (GZ), Yunnan (YN), and Tibet Autonomous Region (XZ). Northwest provinces: Shaanxi (SN), Gansu (GS), Qinghai (QH), Ningxia Hui Autonomous Region (NX), and Xinjiang Uygur Autonomous Region (XJ).

The rate of HIV/HBV/HCV co-infection among HIV/HCV co-infection was 14.9% (32/215) in 2015 and 8.8% (14/158) in 2023. The rates of HIV/HBV co-infection are indicated in Figure 1 and Figure 2, with classification according to transmission modes and regions. For 2015 and 2023, 4501 and 6523 eligible observations are included, respectively. The rates were 13.8% (620/4501) in 2015 and 9.0% (590/6523) in 2023 (Table 2).

Specifically, HIV/HBV co-infection rates declined in the sexual transmission population in 2023 compared to those in 2015 (heterosexual transmission: 10.5% vs. 13.6%, homosexual transmission: 5.9% vs. 13.7%, *p* < 0.05 for both) (Figure 1). Regionally, the rates also declined in North China (5.1% vs. 9.7%), Northeast China (6.1% vs. 10.1%), East China (7.9% vs. 15.8%), Central China (10.1% vs. 13.4%), and South China (11.5% vs. 40.8%) in 2023 (Figure 2, *p* < 0.05 for all). No significant differences were observed in Northwest China (6.7% in 2023 vs. 6.3% in 2015) and Southwest China (10.8% in 2023 vs. 9.2% in 2015), respectively.

### 3.3. Prevalence of HIV/HCV Co-Infection in China

The rate of HBV/HIV/HCV co-infection among HIV/HBV co-infection was 5.2% (32/620) in 2015 and 2.4% (14/590) in 2023. Among newly diagnosed HIV-positive subjects, the rates of HIV/HCV co-infection significantly decreased in 2023 compared to those in 2015 (2.4% vs. 4.8%) (Figure 1 and Table 2). Among HIV/HCV co-infected individuals in 2023, HCV RNA was detected in nearly half of them (49.4%, 78/158).

Different from HIV/HBV co-infection, declined HIV/HCV co-infection was only observed in the heterosexual transmission population (2.5% vs. 4.8%, *p* < 0.05) but not the homosexual transmission population (1.4% vs. 1.9%, *p > 0*.05) in 2023 in comparison to that in 2015. Regionally, the rates of HIV/HCV co-infection were found to be decreased in Northwest China (3.8% vs. 11.3%), South China (1.3% vs. 6.6%), Central China (1.8% vs. 5.9%), and Southwest China (3.3% vs. 6.0%) in 2023 (*p* < 0.05), while no declination was found in North China (1.2% vs. 2.4%), Northeast China (1.5% vs. 3.7%), East China (1.5% vs. 3.7%), and Eastern China (2.0% vs. 1.5%) with a *p*-value over 0.05 (Figure 1 and Figure 2), though a declining trend was observed in most of these regions.

### 3.4. Risk Factors for HIV/HBV and HIV/HCV Co-Infection in China

The univariate logistic regression model study results indicated that age, literacy level, marital status, HIV infection route, and initial CD4+ T-lymphocyte count were the factors impacting the co-infection of HIV/HBV among newly diagnosed HIV-infected subjects in 2023. Multifactorial logistic regression model analysis revealed that among newly diagnosed HIV-positive individuals in 2023, illiteracy and schooling at elementary (AOR = 2.20, 95% CI: 1.60–3.03), middle (AOR = 2.11, 95% CI: 1.54–2.88), and high school levels (AOR = 1.88, 95% CI: 1.32–2.66), heterosexual transmission (AOR = 1.36, 95% CI: 1.07~1.73), and IDU (AOR = 2.91, 95% CI: 1.22~6.93) were statistically associated with the co-infection of HBV (Table 3).

The univariate logistic regression model study results indicate that age, ethnicity, literacy, marital status, and HIV infection pathway were the factors impacting the co-infection of HIV/HCV among newly diagnosed HIV-infected subjects in 2023. Multifactorial logistic regression analysis results revealed a statistically significant association with age 35–49 (AOR = 2.03, 95% CI: 1.29–3.21), other ethnicities (AOR = 2.78, 95% CI: 1.93–4.01), and IDU (AOR = 29.61, 95% CI: 13.01–67.38) (Table 3).

## 4. Discussion

In this study, the rate of HIV/HBV co-infection among newly diagnosed HIV-positive subjects was 9.0% in 2023, which was significantly lower than that in 2015 (13.8%). It is comparable to the rate of HIV/HBV co-infection among HIV-positive subjects receiving free antiretroviral therapy that was reported in China between 2010 and 2012 in China (8.7%, 2958/33,861) [8] but higher than that in the Eastern Mediterranean and Southeast Asia (3.0%), as well as the average of a global pooled analysis (7.0%) of 23 studies that included HIV/HBV coinfections in 11 countries from 1991 to 2018. However, it is still much lower than that in the Western Pacific region (27.0%) [10].

Among newly diagnosed HIV-positive individuals, stratified analyses revealed that a significant decline in HIV/HBV co-infection was observed in heterosexual transmitted and homosexual transmitted populations in 2023 than in 2015. As previously reported, the rate of HIV/HBV co-infection in subjects with heterosexual and homosexual transmission was estimated at 11.3% and 9.6% during 2010–2019 [11]. The data indicate a noteworthy decline in the rate of HBV infection among HIV-positive patients in China for sexual transmission, with a greater decline observed in MSM. This decline may be attributed to the adoption of condom use guidelines for MSM and the fact that MSMs are primarily young individuals in China who have a high rate of hepatitis B vaccination [12]. Our research also revealed that all other regions had significant decreases in HBV co-infection, except the Southwest and Northwest, which showed no decline. Newborns have received the hepatitis B vaccine for free since 2005 in China, especially in the Southeast region where there is high income, and there has been no policy support for free hepatitis B vaccines for adults until now. People who were initially immunized with free hep-B vaccines were already older than 18 years old, and those in the 18–34 age group had already begun to benefit, with significantly lower HIV/HBV co-infection rates than those in the older age group. This might be ascribed to the lower rate of hepatitis B vaccination [12], the greater percentage of heterosexual HIV infection, more rural locations, and lower income levels in the Southwest and Northwest regions. This also suggests that the prevalence of HBV co-infection varies greatly geographically in China. Therefore, it is necessary to strengthen the screening of HBV prevalence in the Southwest and Northwest of China to prevent the spread of further co-infection, which will challenge the prevention and control strategies of both viruses.

This study revealed that the transmission route and literacy level were risk factors for HIV/HBV co-infection. The likelihood of HIV/HBV co-infected subjects increased with lower literacy levels, partially ascribing to low rates of hepatitis B vaccination and low levels of HBV knowledge [12,13]. Injection drug use and heterosexual transmission were also risk factors for HIV/HBV-coinfected patients, which is similar to previous reports and meta-analyses [14]. HBV/HIV co-infection is transmitted more through heterosexual (10.5%) than homosexual (5.9%) transmission. Therefore, in order to better prevent and control HIV/HBV co-infection, it is advised to increase publicization and education in key populations, including low-education individuals, IDUs, and heterosexual individuals who are at higher risk of infection.

According to this study, the percentage of HIV/HCV co-infection in newly diagnosed HIV-positive subjects decreased significantly from 4.8% in 2015 to 2.4% in 2023. The rate of HIV/HCV co-infection in 2023 was higher than that of UK Afro-descendants in 2021 (1.3%) and Ethiopia in 2022 (1.7%) but in line with the 2.4% to 5% worldwide rate during 1991 to 2018 [10]. A notable decline in HIV/HCV co-infection was seen in Spain in 2010 (8.3%) and 2019 (2.2%) [15,16], which is in accordance with the findings in this study, although it is still greater than in high- and middle-income nations like Europe.

Additionally, HIV/HCV co-infection was found to decrease significantly in the heterosexual (4.8% in 2015 vs. 2.5% in 2023) but not homosexual (1.9% in 2015 vs. 1.4% in 2023) transmitted population. This might be ascribed to anal douching [17] and low knowledge about HCV in MSMs, who make up most of the homosexual-transmitted population [18,19]. Due to the baseline rate of HIV/HCV co-infection in MSMs being low, more research is necessary to explore if HIV/HCV co-infection will continue to rise among MSMs. Also, in this study, HIV/HCV co-infection was found to decline dramatically in the Central, South, Southwest, and Northeast China. This may be associated with the use of DAAs to treat HCV infection in China from 2018. The potential reasons for these declines need to be further studied.

According to this study, injectable drug usage, ages 35–49, and ethnic minority status are risk factors for HIV/HCV co-infection. It is possible that unsafe sexual practices, such as prostitution or fornication, are major causes of the increased prevalence of HCV co-infection among elderly HIV-positive individuals. Some studies have shown that ethnic minorities and injecting drug users have been identified as high-risk factors for HCV transmission [14,20,21]. It is suggested that training, publicity, and education might be provided to these key populations with an emphasis on the timely screening of HCV presence.

In conclusion, there has been a considerable decline in the rate of HIV/HBV co-infection among newly diagnosed HIV-positive subjects throughout China. In addition, the rate of HIV/HBV co-infection is greater in the southwest and northwest areas, where HIV/HBV co-infection rate has shown no decline. The percentage of HIV/HCV co-infection in newly diagnosed HIV-positive individuals has also decreased significantly. HIV/HBV co-infected subjects should be given antiretroviral therapy with the TDF regimen, and HIV/HCV co-infected subjects should be given DAAs for hepatitis C, and HIV-positive subjects without HBV co-infection should be given HBV vaccination. All these measures can strengthen the prevention and control of HIV/HBV/HCV transmission.

This study has some limitations. Our study targets HIV-infected subjects and excludes AIDS subjects. The sociodemographic indicators utilized in the research revealed certain data absent, The comparison created between non-absent and absent values for each sociodemographic variable showed no significant shift in Supplementary Table S1. We were unable to identify conditions such as acute HBV or HBV staging. Therefore, the infection rate of HBV may be overestimated.

## Figures and Tables

**Table 1:** Demographic characteristics of newly diagnosed HIV-infected subjects in China in 2015 and 2023.

Characteristic	2015, n (%)	2023, n (%)	*p*	Total, n (%)
Total	4501 (100.0)	6523 (100.0)		11,024 (100.0)
Age (years)			<0.001	
18–29	2304 (51.2)	2082 (31.9)		4386 (39.8)
30–49	1301 (28.9)	1397 (21.4)		2698 (24.5)
≥50	896 (19.9)	3044 (46.7)		3940 (35.7)
Sex			<0.001	
Male	3611 (80.2)	4924 (75.5)		8535 (77.4)
Female	824 (18.3)	1427 (21.9)		2251 (20.4)
Unknown	66 (1.5)	172 (2.6)		238 (2.2)
Route of HIV infection			<0.001	
HET	2185 (48.6)	4246 (65.1)		6431 (58.3)
MSM	1937 (43.0)	2067 (31.7)		4004 (36.3)
IDU	73 (1.6)	33 (0.5)		106 (1.0)
Others	148 (3.3)	104 (1.6)		252 (2.3)
Unknown	158 (3.5)	73 (1.1)		231 (2.1)
Ethnicity			<0.001	
Han	3823 (84.9)	5529 (84.7)		9352 (84.8)
Others	606 (13.5)	976 (14.9)		1582 (14.4)
Unknown	72 (1.6)	18 (0.4)		90 (0.8)
Education			<0.001	
Illiteracy or primary school	940 (20.9)	2353 (36.1)		3293 (29.9)
Junior middle school	1457 (32.4)	1738 (26.7)		3195 (29.0)
High school	910 (20.2)	870 (13.3)		1780 (16.1)
College and above	1114 (24.7)	1456 (22.3)		2570 (23.3)
Unknown	80 (1.8)	106 (1.6)		186 (1.7)
Marital status			<0.001	
Single	1841 (40.9)	2136 (32.7)		3977 (36.1)
Married	1834 (40.7)	2818 (43.2)		4652 (42.2)
Divorced or widowed	728 (16.2)	1460 (22.4)		2188 (19.8)
Unknown	98 (2.2)	109 (1.7)		207 (1.9)
CD4 (cells/μL)			<0.001	
200–350	1548 (34.4)	3260 (50.0)		4808 (43.6)
350–500	1393 (30.9)	1812 (27.8)		3205 (29.1)
>500	1133 (25.2)	1262 (19.3)		2395 (21.7)
Unknown	427 (9.5)	189 (2.9)		616 (5.6)
Administrative region			<0.001	
North	554 (12.3)	572 (8.8)		1126 (10.2)
Northeast	483 (10.7)	329 (5.0)		812 (7.4)
East	1041 (23.1)	1455 (22.3)		2496 (22.6)
Central	764 (17.0)	828 (12.7)		1592 (14.4)
South	348 (7.8)	529 (8.1)		877 (8.0)
Southwest	914 (20.3)	2232 (34.2)		3146 (28.5)
Northwest	397 (8.8)	578 (8.9)		975 (8.9)

Abbreviation: HIV = human immunodeficiency virus; HET = heterosexual; MSM = men who have sex with men; IDU = injection drug use.

**Table 2:** HBV and HCV co-infection in newly diagnosed HIV-positive individuals in 2015 and 2023.

	2015, n (%)	2023, n (%)	*p*	Total, n (%)
HIV/HBV	13.8 (620/4501)	9.0 (590/6523)	<0.001	1210 (11.0)
HIV/HCV	4.8 (215/4501)	2.4 (158/6523)	<0.001	373 (3.3)
HIV/HBV/HCV	0.4 (32/4501)	0.2 (14/6523)	<0.001	46 (0.4)

*p* values were calculated using the χ2 test. *p* values < 0.05 are statistically significant.

**Table 3:** Risk factors of HBV and HCV co-infection in newly diagnosed HIV-positive individuals in China in 2023.

Characteristic	HIV	HBV+ (n, %)	OR (95%CI)	*p*	AOR (95%CI)	*p* *	HCV+ (n, %)	OR (95%CI)	*p*	AOR (95%CI)	*p* *
Age (years)											
18~34	2082	130 (6.2)	1.00				37 (1.8)	1.00		1.00	
35~49	1397	145 (10.4)	1.74 (1.36~2.23)	<0.001			53 (3.8)	2.18 (1.42~3.34)	<0.001	2.03 (1.29~3.21)	0.002
≥50	3044	315 (10.4)	1.73 (1.40~2.14)	<0.001			68 (2.2)	1.26 (0.84~1.89)	0.258	1.45 (0.91~2.32)	0.123
Sex											
Male	4924	428 (8.7)	1.00				113 (2.3)	1.00			
Female	1427	144 (10.1)	1.18 (0.97~1.44)	0.104			41 (2.9)	1.26 (0.88~1.81)	0.212		
Unknown	172	18 (10.5)	1.23 (0.75~2.02)	0.419			4 (2.3)	1.01 (0.37~2.78)	0.979		
Route of HIV infection											
MSM	2067	123 (6.0)	1.00		1.00		107 (2.5)	1.00		1.00	
HET	4246	446 (10.5)	1.86 (1.51~2.28)	<0.001	1.36 (1.07~1.73)	0.011	30 (1.5)	0.57 (0.38~0.86)	0.007	1.28 (0.81~2.03)	
IDU	33	7 (21.2)	4.26 (1.81~10.00)	0.001	2.91 (1.22~6.93)	0.016	154 (5.5)	32.23 (15.82~65.67)	<0.001	29.61 (13.01~67.38)	
Others	104	8 (7.7)	1.32 (0.63~2.77)	0.468	0.99 (0.46~2.09)	0.968	2 (1.9)	0.76 (0.19~3.12)	0.701	0.96 (0.22~4.14)	
Unknown	73	6 (8.2)	1.42 (0.60~3.33)	0.426	1.13 (0.48~2.68)	0.783	4 (5.5)	2.24 (0.80~6.26)	0.123	2.69 (0.90~8.09)	
Ethnicity											
Han	5529	506 (9.2)	1.00				98 (1.8)	1.00		1.00	
Others	976	82 (8.4)	0.91 (0.71~1.16)	0.451			59 (6.1)	3.57 (2.56~4.96)	<0.001	2.78 (1.93~4.01)	<0.001
Unknown	18	21 (1.1)	1.24 (0.29~5.41)	0.774			1 (5.6)	3.26 (0.43~24.74)	0.253	2.94 (0.37~23.03)	0.306
Education											
College and above	1456	65 (4.5)	1.00		1.00		16 (1.1)	1.00			
Illiteracy or primary school education	2353	265 (11.3)	2.72 (2.05~3.59)	<0.001	2.20 (1.60~3.03)	<0.001	78 (3.3)	3.09 (1.79~5.30)	<0.001		
Junior middle school	1738	178 (10.2)	2.44 (1.82~3.27)	<0.001	2.11 (1.54~2.88)	<0.001	38 (2.2)	2.01 (1.12~3.62)	0.020		
High school	870	76 (8.7)	2.05 (1.45~2.88)	<0.001	1.88 (1.32~2.66)	<0.001	21 (2.4)	2.23 (1.16~4.29)	0.017		
Unknown	106	6 (5.7)	1.28 (0.54~3.04)	0.569	1.09 (0.46~2.61)	0.842	5 (4.7)	4.46 (1.60~12.41)	0.004		
Marital status											
Single	2136	131 (6.1)	1.00				39 (1.8)	1.00			
Married	2818	304 (10.8)	1.85 (1.50~2.29)	<0.001			68 (2.4)	1.33 (0.89~1.98)	0.160		
Divorced or widowed	1460	149 (10.2)	1.74 (1.36~2.22)	<0.001			48 (3.3)	1.83 (1.19~2.80)	0.006		
Unknown	109	6 (5.5)	0.89 (0.38~2.07)	0.789			3 (2.8)	1.52 (0.46~5.00)	0.489		
CD4 (cells/μL)											
200–350	3260	311 (9.5)	1.00				74 (2.3)	1.00			
350–500	1812	168 (9.3)	0.97 (0.80~1.18)	0.754			50 (2.8)	1.22 (0.85~1.76)	0.280		
>500	1262	89 (7.1)	0.72 (0.56~0.92)	0.009			31 (2.5)	1.08 (0.71~1.66)	0.709		
Unknown	189	22 (11.6)	1.25 (0.79~1.98)	0.342			3 (1.6)	0.69 (0.22~2.22)	0.539		
Total	6523	590 (9.0)					158 (2.4)				

Note: HBV+ stands for HBsAg-positive at a patient’s initial diagnosis of HIV detection. HCV+ stands for anti-HCV positive at a patient’s first diagnosis of HIV detection. OR stands for odds ratio; AOR stands for adjusted odds ratio; * represents the *p*-value of the multifactor analysis. *p* values < 0.05 are statistically significant.

## Data Availability

Data are contained within the article.

## Supplementary Materials

The following supporting information can be downloaded at https://www.mdpi.com/article/10.3390/pathogens13050367/s1. Table S1: Changes in the prevalence of HBV and HCV between missing and non-missing values in 2015 and 2023.
