# Supplementary material for: HBV and HCV Co-Infection in Chinese Newly Diagnosed HIV+ Subjects in 2015 and 2023: A Cross-Sectional Study
Source: Pathogens. 2024 Apr 29;13(5):367. doi: 10.3390/pathogens13050367 (PMC11124262; doi:10.3390/pathogens13050367)
Supplement: Supplementary file 1 [file pathogens-13-00367-s001.zip › pathogens-2936966-supplementary.pdf]

SUPPLEMENTARY TABLE S1. Changes in the prevalence of HBV and HCV between missing and non-missing values in 2015 and 2023.

**Characteristic**		**2015, n (%)**	**2023, n (%)**	**P**	**Total, n (%)**
Total	Missing or not	4501 (100.0)	6523 (100.0)	<0.001	11024 (100.0)
Sex	No	4435 (98.5)	6351 (97.4)	<0.001	10786 (97.8)
	Yes	66 (1.5)	172 (2.6)	<0.001	238 (2.2)
Route of HIV infection	No	4343 (96.5)	6450 (98.9)	<0.001	10793 (97.9)
	Yes	158 (3.5)	73 (1.1)	<0.001	231 (2.1)
Ethnicity	No	4429 (98.4)	6505 (99.6)	<0.001	10934 (99.2)
	Yes	72 (1.6)	18 (0.4)	<0.001	90 (0.8)
Education	No	4421 (98.2)	6417 (98.4)	<0.001	10838 (98.3)
	Yes	80 (1.8)	106 (1.6)	<0.001	186 (1.7)
Marital status	No	4403 (97.8)	6414 (98.3)	<0.001	10817 (98.1)
	Yes	98 (2.2)	109 (1.7)	<0.001	207 (1.9)
CD4 (cells/μl)	No	4074 (90.5)	6334 (97.1)	<0.001	10408 (94.4)
	Yes	427 (9.5)	189 (2.9)	<0.001	616 (5.6)

P values were calculated using Cochran-Armitage trend test. P values <0.05 is statistically significant.
